# Analysis of SCA8, SCA10, SCA12, SCA17 and SCA19 in patients with unknown spinocerebellar ataxia: a Thai multicentre study

**DOI:** 10.1186/s12883-015-0425-y

**Published:** 2015-09-15

**Authors:** Lulin Choubtum, Pirada Witoonpanich, Suchat Hanchaiphiboolkul, Roongroj Bhidayasiri, Onanong Jitkritsadakul, Sunsanee Pongpakdee, Suppachok Wetchaphanphesat, Pairoj Boonkongchuen, Teeratorn Pulkes

**Affiliations:** Division of Neurology, Department of Medicine, Faculty of Medicine, Ramathibodi Hospital, Mahidol University, Bangkok, Thailand; Research Center, Faculty of Medicine, Ramathibodi Hospital, Mahidol University, Bangkok, Thailand; Department of Neurology, Prasat Neurological Institute, Bangkok, Thailand; Chulalongkorn Center of Excellence on Parkinson Disease and Related Disorders, Department of Medicine, Faculty of Medicine, Chulalongkorn University and King Chulalongkorn Memorial Hospital, Thai Red Cross Society, Bangkok, 10330 Thailand; Division of Medicine, Bhumibol Adulyadej Hospital, Bangkok, Thailand; Division of Neurology, Department of Medicine, Buriram Hospital, Buriram, Thailand

## Abstract

**Background:**

About 50 % of Thai patients with adult-onset spinocerebellar ataxia (SCA) was Machado-Joseph disease (MJD), SCA1, SCA2 and SCA6. The author investigated further on less common SCAs in the patients without any known mutations.

**Methods:**

DNA samples of 82 index patients who were genetically excluded MJD, SCA1, SCA2, SCA6, SCA7 and dentatorubro-pallidoluysian atrophy (DRPLA) were examined. Analysis of SCA8, SCA10, SCA12, SCA17 and SCA19 genes were comprehensively performed. Normal range of trinucleotide repeat expansion sizes of TATA-box-binding protein gene (*TBP*) were also determined in 374 control subjects.

**Results:**

Eight patients carried ≥42 CAG/CAA repeat allele in the *TBP* consistent with SCA17. The pathological repeat alleles ranged from 42 to 57 repeats. All patients had significant degree of cognitive dysfunction. Other non-ataxic phenotypes comprised of parkinsonism, chorea, dystonia and myoclonus. A sporadic patient carried a heterozygous 41-repeat allele developed chronic progressive cerebellar degeneration commenced at the age of 28 years. Whilst, 2 % of the control subjects (8/374) carried the 41-repeat allele. Five of the carriers were re-examined, and revealed that four of them had parkinsonism and/or cognitive impairment without cerebellar signs. Analysis of other types of SCAs was all negative.

**Conclusions:**

This is the first study of SCA8, SCA10, SCA12, SCA17 and SCA19 in Thais. SCA17 appears to be an important cause of ataxia in Thailand. Although, the pathological cut-off point of the *TBP* repeat allele remains unclear, the finding suggests that the 41-repeat may be a pathological allele resulting late-onset or mild phenotype. Apart from ataxia, cognitive impairment and parkinsonism may be clinical presentations in these carriers.

**Electronic supplementary material:**

The online version of this article (doi:10.1186/s12883-015-0425-y) contains supplementary material, which is available to authorized users.

## Background

Spinocerebellar ataxia (SCA) is inherited neurodegenerative disorders in associated with progressive cerebellar degeneration affecting worldwide populations. Causative genetic defects underlying SCA are extensively heterogeneous, which types and prevalence of SCA are widely varied among different populations. In Thailand, almost 90 % of familial SCA families, and over 30 % of sporadic cases are Machado-Joseph disease (MJD, or SCA3), SCA1, SCA2 and SCA6 [1]. In contrast, SCA7 and dentatorubro-pallidoluysian atrophy (DRPLA) have not been observed to date. Since some other uncommon types of SCA have been described in multiple ethnics, those certain types are merit for further screening in the Thai patients [2]. The study was interested in investigating some uncommon SCAs including SCA8, SCA10, SCA12, SCA17 and SCA19.

SCA8 is caused by bidirectional transcribed of CTG-CAG repeat-expansion mutation of ataxin 8 opposite strand (*ATXN8OS*), and ataxin 8 (*ATXN8*) on chromosome 13q21 [3]. The disease is transmitted in autosomal dominant fashion with often reduced penetrance [4]. Patients present as a slowly progressive cerebellar syndrome frequently accompanied with pyramidal signs [4, 5]. SCA10 is an autosomal dominant ataxia often accompanied with epilepsy caused by an expansion of a pentanucleotide (ATTCT) repeat in intron 9 of *ATXN10*. The disease is exclusively identified in Amerindian descents mainly in South America, in which they are likely to share a common ancestral origin [6, 7]. SCA12 is caused by a CAG repeat expansion in a promoter region of the *PPP2R2B* on chromosome 5q32 [8]. Action tremor is often a prominent feature. Apart from Caucasians, SCA12 is also identified as an infrequent cause of SCA in Indians and Chinese [9–11]. SCA17 is caused by an abnormal CAG/CAA repeat expansion in TATA-binding protein gene (*TBP*) [12]. Various additional features including parkinsonism, chorea, dystonia and dementia is common. Although SCA17 regards as an uncommon type of SCA, the disease is described in quite a few populations including in East Asian [13, 14]. Furthermore, the pathological repeat-expansion alleles of SCA17 were also observed in association with Parkinson’s disease (PD) in Taiwanese and Korean, implying the occurrence of SCA17 might be relatively common in East Asian [15, 16]. SCA19 and SCA22 were originally described in Dutch and Han Chinese families, respectively. Both diseases were mapped to the same locus on chromosome 1q [17]. Their phenotypes were similar in some extents, which they often present as a very slowly progressive ataxia with frequent hyporeflexia. However, additional features including Holmes tremor, myoclonus and impairment of proprioceptive sensation were only observed in some cases of the Dutch family [18, 19]. Recently, a voltage-gated potassium channel Kv4.3-encoding gene (*KCND3*) was described as a causative gene responsible for both SCA19 and SCA22 [20, 21].

The study attempted to identify the SCA subtypes in Thai patients without any known genetic mutations, and defined their genotype-phenotype correlations. SCA8, SCA12, SCA17, and SCA19/22 were designated to analyse because they were described in multiple populations. Since a few of the studied patients had epilepsy as an additional feature, SCA10 was designated to investigate as a candidate gene.

### Methods

#### Participants and DNA samples

We studied on all collected DNA samples of patients with progressive cerebellar degeneration, in which genetic tests for SCA1, SCA2, MJD, SCA6, SCA7 and DRPLA were negative. The studied patients were also carefully investigated to exclude other possible diagnoses including autoimmune cerebellar disorders, cerebellar variant of multiple system atrophy, and common mitochondrial DNA mutations by using appropriated blood tests, genetic analysis of leukocyte, or muscle DNA and neuroimaging. DNA samples of 82 index patients were available for eventually complete all genetic analyses in the study. All obtainable medical records of the participants were reviewed. Accessible patients were also re-examined. Control subjects were individuals aged over 65 years old without signs of any movement disorders. The control DNA samples were previously obtained for previous study on Parkinson genetics during 2008 to 2013 [22].

#### Ethics, consent and permissions

The research protocol was approved by the ethics committee of the Ramathibodi Hospital, Mahidol University (ID 03-53-19). All participants were provided appropriate genetic counselling and informed consent prior taking their blood samples.

#### Genetic analysis

##### Size of repeat expansion

DNA samples were extracted from peripheral leukocyte by phenol-chloroform method or using QIAGEN DNA purification kit (QIAGEN, CA, USA). Fluorescent PCR of the tandem repeat alleles of the *ATXN8OS* (SCA8)*, PPP2R2B* (SCA12) and *TBP* (SCA17) were undertaken by using methods kindly provided by Neurogenetics Lab, UCL Institute of Neurology, London. The PCR amplicons were checked on agarose gel. The allele sizes were then determined by capillary electrophoresis on the 3730XL DNA Analyzer (Applied Biosystems, CA, USA). PCR fragments were analysed by using Peak Scanner V.1 (Applied Biosystems, CA, USA). Sizes of tandem repeat of both normal and pathological alleles of each chromosome were calculated by comparing results with the size of normal control samples, of which the sizes were prior defined by direct sequencing. Sizes of normal alleles in Thai individuals were determined by analysis in 80 controls for SCA8 and SCA12 alleles, and 374 control subjects for SCA17 allele. Identification of normal and abnormal repeat expansion alleles for each SCA subtypes were established by using data of previous reports, which they were considered as the following circumstances: normal SCA8 allele ranged from 15 to 50 repeats [23]; normal SCA12 allele ranged from 4 to 32 repeats [24]; and pathological SCA17 alleles were ≥ 42 repeats [16].

#### Fluorescent repeat-primed PCR assay

*ATXN10* (SCA10) was screened by fluorescent repeat-primed PCR assay [25]. SCA10 is associated with a huge, several hundred of pentanucleotide repeats, while sizes of normal alleles are generally very short with upper limit of 33 repeats. Under this circumstance, fluorescent repeat-primed PCR amplification is a simple screening method, which is able to distinguish a normal short tandem repeats from potentially pathological SCA10 expansion alleles [25]. Electrophoresis was run on the 3730XL DNA Analyzer, and analysis of the PCR fragments were determined using Peak Scanner V.1.

#### Sequencing analysis

All 8 exons and exon-intron boundaries of *KCND3* (NT019273; NM004980) were directly sequenced on both strands in all patients by using the Big Dye Terminator Cycle Sequencing kit (Applied Biosystems, CA, USA). Primer sets and PCR conditions (shown in Additional file 1: Table S1) were newly designed according to the standard *KCND3* sequence [26]. The PCR products were then loaded into the 3730XL DNA Analyzer and analysed with the Sequence Analysis software v3.0 (Applied Biosystems, CA, USA).

#### Statistical analysis

Results were expressed as means ± standard deviations. Correlation between trinucleotide repeat lengths and age at onset were determined by linear regression model. The *p*-value associated with the correlation coefficient (*r*) was calculated on the basis of two-tailed probabilities. *P* less than 0.05 was considered to be statistical significant. Analysis was performed on VassarStats website for statistical computation (http://www.vassarstats.net).

## Results

The study identified only SCA17 mutation in this panel of 82 unrelated Thai patients with cerebellar degeneration of unknown origin. There were eight patients having ≥ 42 trinucleotide repeat alleles in the *TBP*. Five of these eight patients had complete medical records, and four patients were also re-examined. Available clinical details of the SCA17 patients are shown in Table 1. Only one patient had family history compatible with autosomal dominant inheritance, while the rest of the patients were sporadic. However, we did not examine parents and other family members of those patients. Phenotypes were quite variable including (1) spinocerebellar ataxia accompanied with prominent parkinsonism; (2) spinocerebellar ataxia accompanied with generalized chorea, dystonia and myoclonus; (3) relatively pure cerebellar syndrome. Dementia was common feature in association with all cases. Gazed-evoked nystagmus, broken pursuit and saccadic abnormalities also sometimes observed. Mean age at onset was 34.4 ± 16.1 years (range 18–67 years). Age at onset was relatively earlier in the patients carrying over 50 repeat alleles than in the patients carried smaller pathological alleles. Linear regression analysis demonstrated a reverse correlation between the sizes of trinucleotide repeats and age at onset with the correlation coefficient (*r*) of −0.84 [*r*^*2*^ = 0.71; *p* = 0.017, 95 % confidence interval = (−0.98) - (−0.25)]. The regression line suggested that an expected age at onset was decreased for an interval of 2.173 years on the basis of one repeat expansion changed. Nevertheless, this formula could not predict an accurate age at onset for each particular patient.

**Table 1:** Clinical details of SCA17 patients

Patient No.	Age at onset (years)	Duration (years)	Gender	Family history	Severity of ataxia	Additional features	Pathological CAG/CAA repeat length
32	24	2	M	no	severe	parkinsonism, foot dystonia, pyramidal signs, dementia	54
47	26	9	M	no	severe	parkinsonism, dementia	54
19.1	na	na	M	na	na	na	43
39.3	40	1	F	no	mild	none^a^	42
128	36	12	M	AD	severe	generalized chorea, limb dystonia, myoclonus, dementia	45
154	30	4	F	no	moderate	na	52
179	18	2	M	no	moderate	na	57
259	67	7	F	no	mild	neck tremor, mild cognitive impairment	42

*M* male, *F* female, *AD* autosomal dominant, *na* not available

^a^The patient was not assessed by adequate cognitive evaluation

Cognitive impairment was the most common additional feature identified in most patients examined. Three out of four examined patients had dementia and one had mild cognitive impairment involving both executive and memory functions. The other patient (no. 39.3) did not have an adequate cognitive assessment in her medical record. The three remaining patients were lost to follow up; they were only known to have progressive cerebellar degeneration with ataxia. Cognitive impairment developed even only a few years after the onset, which it could progress to severe dementia at very young age. Similar to previous reports, parkinsonism, dystonia and chorea were additional non-ataxic features observed in SCA17 patients.

**Fig. 1:**
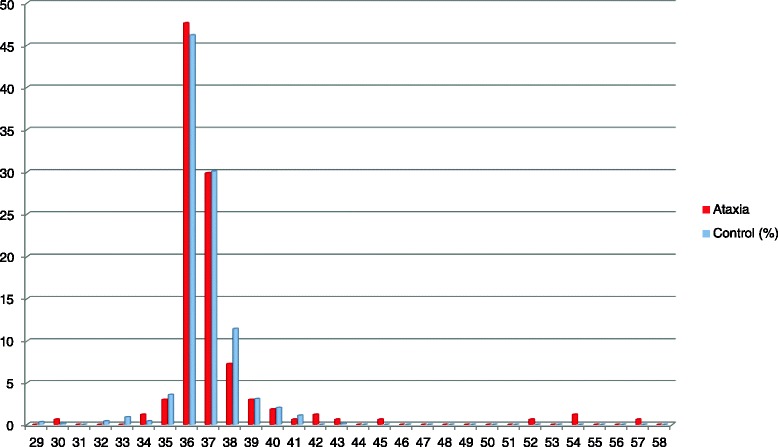
Frequency distribution of CAG/CAA repeat lengths in ataxia and healthy control groups

Association between a heterozygous 41-repeat allele of *TBP* (SCA17) and phenotypic expression has remained unclear. Therefore the study presented the actual observations related to the carriers of the *TBP* 41-repeat allele without a definite conclusion. Mean of the tandem repeat sizes of *TBP* in 374 control subjects was 37.12 ± 3.12 repeats (mode = 36; median = 36) (Fig. 1). Only one female patient carried a heterozygous 41 repeats (allele frequency = 0.006), whereas eight control subjects (allele frequency = 0.01) did. The 29-year-old female patient has developed progressive ataxia for one year. Her phenotype was pure cerebellar ataxia. She had no family of any movement disorders. The other carriers of the 41-repeat allele (control group) aged from 66 to 81 years old (mean 74 ± 4.5 years). Two of these controls were unable to be contacted, and one of them was deceased. Thus, we re-examined five out of the eight carriers. Examination revealed no cerebellar signs in all carriers. However, three of them had mild, but definite signs of parkinsonism with typical rest tremor in one carrier. Two of these patients also had significant cognitive impairment [Montreal Cognitive Assessment (MoCA) = 13 and 18]. One of the carriers without parkinsonism, a 66-year-old female, had mild cognitive impairment (MoCA = 23). The remaining patient was in terminal stage of cirrhosis without any signs of parkinsonism.

Genetic analyses for SCA8, SCA10, SCA12 and SCA19/22 were all unremarkable. Mean of the tandem repeat sizes of *ATXN8OS* (SCA8) was 23.07 ± 3.94 repeats (mode = 18; range = 18–32 repeats), and mean of the tandem repeat sizes of *PPP2R2B* (SCA12) was 13.14 ± 2.60 repeats (mode = 10; range = 9–24 repeats). Sequencing of *KCND3* in all of the patients was identified only heterozygous single nucleotide polymorphisms including one newly identified silent variant in exon 2 (p.Pro88Pro), one known silent variant (p.Pro125Pro; rs2289723), and three intron variants (rs369047161, rs72548727 and rs640029).

## Discussion

Spinocerebellar ataxias are widely heterogeneous neurodegenerative disorders, which definitive diagnosis is often required DNA analysis using various specific genetic testings. To set-up the optimal genetic tests for certain population are often needed to rely on its own epidemiological data. Information regarding subtypes of SCA in Thai population has still limited. Analysis of the common SCA subtypes revealed that about 50 % of the patients were MJD, SCA1, SCA2 and SCA6 [1]. In an effort to further describe the specific SCA genotypes in the remaining patients without any known cause, the study identified that about 10 % of these patients carried SCA17 mutation. Whilst SCA8, SCA10, SCA12 and SCA19 were absent in the study implying that they appear to be rare in Thai population.

SCA17 is relatively uncommon, despite its worldwide distribution. For instance, SCA17 accounted for only 1 % of the SCA patients in a large Caucasian cohort [27]. Most reports described only single, or a few families. To date, less than 100 families have been described [28]. This study cohort was originally consisted of 265 families with ataxia [1]. About half of those SCA families were unable to determine cause. Although only 82 DNA samples (63 % of the remaining patients with unknown cause) were available for the study, the authors identified 8 unrelated patients (~10 %) carried CAG/CAA expansion of the *TBP* gene. This data suggests that SCA17 is an important cause of adult-onset progressive ataxia in Thais especially coexisted with dementia, parkinsonism and chorea, despite sporadic nature.

Phenotype of SCA17 is often variable. It frequently accompanies with dementia and other abnormal movements including parkinsonism, chorea, dystonia and myoclonus [12, 27, 29–31]. Phenotypes of Thai SCA17 patients in the study were similar to those prior described in Caucasians and Japanese. Since the previous work in the large Thai cohort demonstrated that all these features particularly abnormal movements were extremely uncommon in association with MJD, SCA1, SCA2, and SCA6 [1]. Those signs especially parkinsonism and dementia is quite useful for helping on differential diagnosis between SCA17 and those common SCAs in Thais.

Sizes of the SCA17 triplet repeat appear to have correlate to some clinical expressions. SCA17 can also exhibit Huntington’s disease-like (HDL) phenotype [30, 32]. The phenotype is often observed in association with a moderately small pathological repeat size, or generally less than 50 repeats. Likewise, the only studied case (No. 128) having generalized chorea and dementia resembling HDL had the repeat size of 45, and none of the patients carried the repeat size greater than 50 developed chorea. Furthermore, the study observed the significant inverse correlation between the sizes of CAG/CAA repeat and age at onset of the disease. The repeat expansion greater than 50 often associated with an early age at onset before 30 years. The latest age at onset was 67 in a female patient (No.259) carried a 42-repeat allele. This observed correlation is in line with some previous reports [27].

Parkinsonism and dementia is one of the most common non-ataxic features in association with SCA17. Moreover, the repeat expansions of SCA17 were formerly detected in a small proportion of patients with PD [15]. Those patients often carried a relatively small SCA17 pathological alleles (43–46 repeats). Even asymptomatic carriers of the 42-repeat allele had decreased striatal DAT binding implying that the expansion as low as 42 repeats might contribute to develop parkinsonism [16]. In the study, one female patient, and eight of the control subjects carried the 41-repeat SCA17 allele. Although ataxia was not observed in the control subjects, parkinsonism and cognitive impairment was clearly evident in almost all control subjects after re-examined. To our knowledge, there were two earlier case reports of SCA17 associated with the 41-repeat allele [33, 34]. Our data together with the others suggest that the 41 CAG/CAA repeat of the SCA17 gene may not be a neutral polymorphisms, but it likely contributes to a wide range of neurodegeneration of the striatal, cognitive and cerebellar pathway.

Most of the studied SCA17 patients were sporadic cases. The lack of family history in the study may explain by several factors. Firstly, it was a retrospective study; some important information might be missing. Alternatively, the patients’ parents might remain asymptomatic due to anticipation [35]. Or, it might cause by that SCA17 was transmitted by an autosomal dominant fashion with reduce penetrance [12, 29, 30].

## Conclusion

In summary, the study demonstrates that SCA17 is an important cause of adult-onset spinocerebellar ataxia in Thailand. In contrast, SCA8, SCA10, SCA12, and SCA19 are rare. Parkinsonism and cognitive impairment are useful for clinically distinguishing between SCA17 and other common SCA subtypes in Thai patients. Although the cut-off point of the pathological CAG/CAA expansion of the SCA17 gene remains unclear, the study provides evidence that the 41 repeat allele is likely to contribute some pathogenic effect. Carriers of the 41 repeat of the SCA17 gene may need a long-termed follow up.

## Additional file

Additional file 1: Table S1. Primer sets and annealing temperatures for *KCND3* sequencing. (DOCX 17 kb)

## Abbreviations

SCA: Spinocerebellar ataxia
MJD: Machado-Joseph disease
DRPLA: Dentatorubro-pallidoluysian atrophy
*ATXN8OS*: Ataxin 8 opposite strand
*ATXN*8: Ataxin 8
*ATXN*10: Ataxin 10
*TBP*: TATA-binding protein gene
*KCND3*: Voltage-gated potassium channel Kv4.3-encoding gene
*r*: Correlation coefficient

## References

[1] Boonkongchuen P, Pongpakdee S, Jindahra P, Papsing C, Peerapatmongkol P, Wetchaphanphesat S, Paiboonpol S, Dejthevaporn C, Tanprawate S, Nudsasarn A (2014). Clinical analysis of adult-onset spinocerebellar ataxias in Thailand. BMC Neurol.

[2] Klockgether T, Paulson H (2011). Milestones in ataxia. Mov Disord.

[3] Moseley ML, Zu T, Ikeda Y, Gao W, Mosemiller AK, Daughters RS, Chen G, Weatherspoon MR, Clark HB, Ebner TJ (2006). Bidirectional expression of CUG and CAG expansion transcripts and intranuclear polyglutamine inclusions in spinocerebellar ataxia type 8. Nat Genet.

[4] Koob MD, Moseley ML, Schut LJ, Benzow KA, Bird TD, Day JW, Ranum LP (1999). An untranslated CTG expansion causes a novel form of spinocerebellar ataxia (SCA8). Nat Genet.

[5] Ikeda Y, Shizuka-Ikeda M, Watanabe M, Schmitt M, Okamoto K, Shoji M (2000). Asymptomatic CTG expansion at the SCA8 locus is associated with cerebellar atrophy on MRI. J Neurol Sci.

[6] Matsuura T, Yamagata T, Burgess DL, Rasmussen A, Grewal RP, Watase K, Khajavi M, McCall AE, Davis CF, Zu L (2000). Large expansion of the ATTCT pentanucleotide repeat in spinocerebellar ataxia type 10. Nat Genet.

[7] Almeida T, Alonso I, Martins S, Ramos EM, Azevedo L, Ohno K, Amorim A, Saraiva-Pereira ML, Jardim LB, Matsuura T (2009). Ancestral origin of the ATTCT repeat expansion in spinocerebellar ataxia type 10 (SCA10). PLoS ONE.

[8] Holmes SE, O’Hearn EE, McInnis MG, Gorelick-Feldman DA, Kleiderlein JJ, Callahan C, Kwak NG, Ingersoll-Ashworth RG, Sherr M, Sumner AJ (1999). Expansion of a novel CAG trinucleotide repeat in the 5′ region of PPP2R2B is associated with SCA12. Nat Genet.

[9] Fujigasaki H, Verma IC, Camuzat A, Margolis RL, Zander C, Lebre AS, Jamot L, Saxena R, Anand I, Holmes SE (2001). SCA12 is a rare locus for autosomal dominant cerebellar ataxia: a study of an Indian family. Ann Neurol.

[10] Zhao Y, Tan EK, Law HY, Yoon CS, Wong MC, Ng I (2002). Prevalence and ethnic differences of autosomal-dominant cerebellar ataxia in Singapore. Clin Genet.

[11] Xie QY, Liang XL, Li XH (2005). Molecular genetics and its clinical application in the diagnosis of spinocerebellar ataxias. Zhonghua yi xue yi chuan xue za zhi.

[12] Koide R, Kobayashi S, Shimohata T, Ikeuchi T, Maruyama M, Saito M, Yamada M, Takahashi H, Tsuji S (1999). A neurological disease caused by an expanded CAG trinucleotide repeat in the TATA-binding protein gene: a new polyglutamine disease?. Hum Mol Genet.

[13] Wang J, Shen L, Lei L, Xu Q, Zhou J, Liu Y, Guan W, Pan Q, Xia K, Tang B (2011). Spinocerebellar ataxias in mainland China: an updated genetic analysis among a large cohort of familial and sporadic cases. Zhong Nan Da Xue Xue Bao Yi Xue Ban.

[14] Maruyama H, Izumi Y, Morino H, Oda M, Toji H, Nakamura S, Kawakami H (2002). Difference in disease-free survival curve and regional distribution according to subtype of spinocerebellar ataxia: a study of 1,286 Japanese patients. Am J Med Genet.

[15] Wu YR, Lin HY, Chen CM, Gwinn-Hardy K, Ro LS, Wang YC, Li SH, Hwang JC, Fang K, Hsieh-Li HM (2004). Genetic testing in spinocerebellar ataxia in Taiwan: expansions of trinucleotide repeats in SCA8 and SCA17 are associated with typical Parkinson’s disease. Clin Genet.

[16] Kim JY, Kim SY, Kim JM, Kim YK, Yoon KY, Kim JY, Lee BC, Kim JS, Paek SH, Park SS (2009). Spinocerebellar ataxia type 17 mutation as a causative and susceptibility gene in parkinsonism. Neurology.

[17] Schelhaas HJ, Verbeek DS, Van de Warrenburg BP, Sinke RJ (2004). SCA19 and SCA22: evidence for one locus with a worldwide distribution. Brain.

[18] Schelhaas HJ, Ippel PF, Hageman G, Sinke RJ, van der Laan EN, Beemer FA (2001). Clinical and genetic analysis of a four-generation family with a distinct autosomal dominant cerebellar ataxia. J Neurol.

[19] Chung MY, Lu YC, Cheng NC, Soong BW (2003). A novel autosomal dominant spinocerebellar ataxia (SCA22) linked to chromosome 1p21-q23. Brain.

[20] Duarri A, Jezierska J, Fokkens M, Meijer M, Schelhaas HJ, den Dunnen WF, van Dijk F, Verschuuren-Bemelmans C, Hageman G, van de Vlies P (2012). Mutations in potassium channel kcnd3 cause spinocerebellar ataxia type 19. Ann Neurol.

[21] Lee YC, Durr A, Majczenko K, Huang YH, Liu YC, Lien CC, Tsai PC, Ichikawa Y, Goto J, Monin ML (2012). Mutations in KCND3 cause spinocerebellar ataxia type 22. Ann Neurol.

[22] Pulkes T, Choubtum L, Chitphuk S, Thakkinstian A, Pongpakdee S, Kulkantrakorn K, Hanchaiphiboolkul S, Tiamkao S, Boonkongchuen P (2014). Glucocerebrosidase mutations in Thai patients with Parkinson’s disease. Parkinsonism Relat Disord.

[23] Ayhan F, Ikeda Y, Dalton JC, Day JW, Ranum LPW. Spinocerebellar Ataxia Type 8. In: Pagon RA, Adam MP, Ardinger HH, Wallace SE, Amemiya A, Bean LJH, Bird TD, Dolan CR, Fong CT, Smith RJH et al editors. GeneReviews(R)*.* edn. University of Washington, Seattle (WA); 1993-2015.

[24] Margolis RL, O’Hearn E, Holmes SE, Srivastava AK, Mukherji M, Sinha KK. Spinocerebellar Ataxia Type 12. In: Pagon RA, Adam MP, Ardinger HH, Wallace SE, Amemiya A, Bean LJH, Bird TD, Dolan CR, Fong CT, Smith RJH et al. editors. GeneReviews(R). edn. University of Washington, Seattle (WA); 1993-2015.

[25] Cagnoli C, Michielotto C, Matsuura T, Ashizawa T, Margolis RL, Holmes SE, Gellera C, Migone N, Brusco A (2004). Detection of large pathogenic expansions in FRDA1, SCA10, and SCA12 genes using a simple fluorescent repeat-primed PCR assay. J Mol Diagn.

[26] Postma AV, Bezzina CR, de Vries JF, Wilde AA, Moorman AF, Mannens MM (2000). Genomic organisation and chromosomal localisation of two members of the KCND ion channel family, KCND2 and KCND3. Hum Genet.

[27] Rolfs A, Koeppen AH, Bauer I, Bauer P, Buhlmann S, Topka H, Schols L, Riess O (2003). Clinical features and neuropathology of autosomal dominant spinocerebellar ataxia (SCA17). Ann Neurol.

[28] Toyoshima Y, Onodera O, Yamada M, Tsuji S, Takahashi H. Spinocerebellar Ataxia Type 17. In: Pagon RA, Adam MP, Ardinger HH, Wallace SE, Amemiya A, Bean LJH, Bird TD, Dolan CR, Fong CT, Smith RJH et al. editors. GeneReviews(R). edn. University of Washington, Seattle (WA); 1993-2015. 20301611

[29] Zuhlke C, Gehlken U, Hellenbroich Y, Schwinger E, Burk K (2003). Phenotypical variability of expanded alleles in the TATA-binding protein gene. Reduced penetrance in SCA17?. J Neurol.

[30] Stevanin G, Fujigasaki H, Lebre AS, Camuzat A, Jeannequin C, Dode C, Takahashi J, San C, Bellance R, Brice A (2003). Huntington’s disease-like phenotype due to trinucleotide repeat expansions in the TBP and JPH3 genes. Brain.

[31] Nakamura K, Jeong SY, Uchihara T, Anno M, Nagashima K, Nagashima T, Ikeda S, Tsuji S, Kanazawa I (2001). SCA17, a novel autosomal dominant cerebellar ataxia caused by an expanded polyglutamine in TATA-binding protein. Hum Mol Genet.

[32] Toyoshima Y, Yamada M, Onodera O, Shimohata M, Inenaga C, Fujita N, Morita M, Tsuji S, Takahashi H (2004). SCA17 homozygote showing Huntington’s disease-like phenotype. Ann Neurol.

[33] Nanda A, Jackson SA, Schwankhaus JD, Metzer WS (2007). Case of spinocerebellar ataxia type 17 (SCA17) associated with only 41 repeats of the TATA-binding protein (TBP) gene. Mov Disord.

[34] Herrema H, Mikkelsen T, Robin A, LeWitt P, Sidiropoulos C (2014). SCA 17 phenotype with intermediate triplet repeat number. J Neurol Sci.

[35] Maltecca F, Filla A, Castaldo I, Coppola G, Fragassi NA, Carella M, Bruni A, Cocozza S, Casari G, Servadio A (2003). Intergenerational instability and marked anticipation in SCA-17. Neurology.

