# Supplementary material for: Analysis of SCA8, SCA10, SCA12, SCA17 and SCA19 in patients with unknown spinocerebellar ataxia: a Thai multicentre study
Source: BMC Neurol. 2015 Sep 15;15:166. doi: 10.1186/s12883-015-0425-y (PMC4571065; doi:10.1186/s12883-015-0425-y)
Supplement: Additional file 1: Table S1. — Primer sets and annealing temperatures for KCND3 sequencing. (DOCX 17 kb) [file 12883_2015_425_MOESM1_ESM.docx]

**Additional file 1: Table S1:** Primer sets and annealing temperatures for *KCND3* sequencing

|  |  | Tm | size |
| --- | --- | --- | --- |
|  |  |  |  |
| KCND3_E2/1_F | GAGACTTTGGCCGTTTCACT | 58 | 588 |
| KCND3_E2/1_R | TTGTACTCCTCGTAGCAGCA |  |  |
|  |  |  |  |
| KCND3_E2/2F | CTTCTTCAACGAGGACACCAAG | 58 | 601 |
| KCND3_E2/2R | ACCGATGTAGTAGGGCATGATG |  |  |
|  |  |  |  |
| KCND3_E2/3F | TACGTGACTGGCTTCTTC | 61 | 664 |
| KCND3_E2/3R | CACTTGGGTAAGGGACTC |  |  |
|  |  |  |  |
| KCND3_E3F | CCAGGTCCATGTCACAGTACC | 65 | 435 |
| KCND3_E3R | TTGCAAAGCCAGAGGCCATC |  |  |
|  |  |  |  |
| KCND3_E4F | AACTGGAGATGCATGAGGTTG | 58 | 420 |
| KCND3_E4R | ATCCTATGGAAGTGGTGTGTG |  |  |
|  |  |  |  |
| KCND3_E5F | TGAGCCCAAAGGTTAGCA | 58 | 365 |
| KCND3_E5R | GGATCTGAAGGGGACAGA |  |  |
|  |  |  |  |
| KCND3_E6F | TTGACTCAAAGGGTGGAATGC | 58 | 286 |
| KCND3_E6R | CAGAAGAATCAGCAGCACATG |  |  |
|  |  |  |  |
| KCND3_E7F | GGCAGCCATACAGAGTATAGG | 58 | 488 |
| KCND3_E7R | TTGGGTTTCCTTGCAAGGAA |  |  |
|  |  |  |  |
| KCND3_E8F | TCCTAGTTACCACGAGCA | 58 | 515 |
| KCND3_E8R | TGCAGTATCACAGGGCTA |  |  |
